# Classification and identification of *Rhodobryum roseum* Limpr. and its adulterants based on fourier-transform infrared spectroscopy (FTIR) and chemometrics

**DOI:** 10.1371/journal.pone.0172359

**Published:** 2017-02-16

**Authors:** Zhen Cao, Zhenjie Wang, Zhonglin Shang, Jiancheng Zhao

**Affiliations:** 1 College of Life Science, Hebei Normal University, Shijiazhuang, China; 2 Hebei College of Industry and Technology, Shijiazhuang, China; Aligarh Muslim University, INDIA

## Abstract

Fourier-transform infrared spectroscopy (FTIR) with the attenuated total reflectance technique was used to identify *Rhodobryum roseum* from its four adulterants. The FTIR spectra of six samples in the range from 4000 cm^−1^ to 600 cm^−1^ were obtained. The second-derivative transformation test was used to identify the small and nearby absorption peaks. A cluster analysis was performed to classify the spectra in a dendrogram based on the spectral similarity. Principal component analysis (PCA) was used to classify the species of six moss samples. A cluster analysis with PCA was used to identify different genera. However, some species of the same genus exhibited highly similar chemical components and FTIR spectra. Fourier self-deconvolution and discrete wavelet transform (DWT) were used to enhance the differences among the species with similar chemical components and FTIR spectra. Three scales were selected as the feature-extracting space in the DWT domain. The results show that FTIR spectroscopy with chemometrics is suitable for identifying *Rhodobryum roseum* and its adulterants.

## Introduction

Mosses are small perennial plants that are typically 1–10 cm tall, and more than 12 000 species have been recognized worldwide [1]. Many moss species have broad geographical locations that span several continents. Mosses commonly concentrate in groups [2]. The persistent photosynthetic phase of the moss life cycle is the gametophyte generation. Spores are released from a sporophyte capsule at certain times [3].

Mosses are one of the most speciose among plants, but few have been used as medical plants. *Rhodobryum roseum* is a medicinal moss species. *Rhodobryum roseum* extractives such as ursolic acid, flavonoids, and alkaloids have been used in cardiac study, and the extracts are more frequently used in medical research [3]. Mosses show extensive morphological and anatomical diversification in both gametophyte and sporophyte organizations [4–6]. Because of the small sizes of mosses, it is difficult to distinguish *Rhodobryum roseum* and its adulterants by using traditional phytotaxonomic methods. In particular, in field collection, *Rhodobryum roseum* is usually confused with *Rhodobryum ontariense* from the same genera and *Plagiomnium actum*, *P*. *maximoviczii*, and *Mnium laevinarve* from adjacent groups. Several modern analytical methods such as molecular biological approaches have been applied to identify the confused moss species. *Rhodobryum Roseum* is easily identified by Random Amplified Polymorphic DNA (RAPD) [7]. However, this method uses large amounts of the material and can be very expensive.

Fourier-transform infrared (FTIR) spectroscopy is an original spectroscopic technique to discriminate various species. FTIR has been used to classify seed plants [8–11], spore plants [12], fungi [13], bacteria [14] and microorganisms [15], and it requires only several milligrams of materials [16]. FTIR has also been used to identify biological tissues [17–19]. Although FTIR spectroscopy has made a significant contribution to plant classification, there are few reports on the identification of mosses using FTIR.

Each FTIR spectrum of a compound can express a unique “fingerprint”, which allows FTIR spectroscopy to be used in the classification of different samples or identification of unknown samples [20]. However, in numerous problems in practice, judgments cannot be rapidly and accurately made by purely relying on the FTIR spectroscopy analysis [9]. Chemometric methods with FTIR spectroscopy can compensate for the errors that occur in a FTIR spectral analysis [21]. Our study aims to discriminate between *Rhodobryum Roseum *and its adulterants by using FTIR spectra with several chemometric methods.

Cluster analysis and principal component analysis (PCA) are two multivariate analyses for identifying the natural clustering pattern and group objects based on the similarities among samples [22]. Cluster analysis is an undirected and unbiased statistical method for analyzing spectroscopic information [23]. Cluster analysis and PCA are widely recognized as powerful tools to obtain information about the relations in a dataset [19]. To classify and compare the spectra of species from different genera, it is necessary to obtain FTIR spectra by using multivariate methods.

Wavelet transform (WT) is another useful tool for various signal-processing applications. WT was also developed to discriminate non-stationary signals with different frequency features [24]. The signal to be analyzed is multiplied by a wavelet function. The analyzing ability for the same signal of wavelet coefficients vary at different scales. Therefore, the WT coefficients can be considered the characteristics of a signal. Several features can reflect the major spectral information after a wavelet function. WT can be considered one of the most efficient chemometric methods [11]. Discrete WT (DWT) is used to decompose a signal by using filters to extract the frequency resolution components of interest in the signal. DWT has compact support in both time and frequency domains [25]. DWT is used to analyze the signal at different frequency bands with different resolutions by decomposing the signal into a coarse approximation and detailed information [26]. This technology is a signal-processing tool that has been used in numerous engineering, scientific, and mathematical applications, and DWT can solve numerous difficult problems that Fourier nsform cannot. Hence, DWT is also known as the "math microscope" [27].

In this study, six samples of mosses were classified: two species of *Rhdobryum roseum* (*Rhdobryum roseum*1 and *Rhdobryum roseum*2) collected from different areas, *Rhodobryum ontariense*, *Plagiomnium actum*, *P*. *maximoviczii*, and *Mnium laevinarve*. The present study aimed to evaluate the potential use of FTIR with an attenuated total reflection (ATR) unit spectroscopy, second-derivative transformation, cluster analysis and PCA to discriminate *Rhdobryum roseum* from the other four confused species of mosses. Fourier self-deconvolution (FSD) and DWT were used to investigate the chemical fingerprint variability among the species.

## Materials and methods

### Species records

In this study, six species of epiphytic bryophytes were collected from Xiao wutai National Nature Reserve (Zhuolu county, Hebei province, China) in August 2011. Xiao wutai National Nature Reserve Administration gave permission for each location of the species. We georeferenced the detailed location information of the 6 species using GPS. The geographic coordinates, altitudes, and sample collection sites are shown in Table 1. The environmental conditions and population sizes of the sample plants were similar. The voucher specimens were deposited in the herbarium of Hebei Normal University.

**Table 1:** Description of the Geographic Coordinates and Altitudes of the Aample Sources.

No.	Family	Genera	Species	Sites	Altitude (m asl)/ Geographic coordinates	Voucher specimen No.
1	Bryaceae	*Rhodobryum*	*Rhodobryum roseum*1	ZL^a^	1309/39°42’N114°38’E	110087X
2			*Rhodobryum roseum*2	ZL^a^	1720/39°56’N115°73’E	110419
3			*Rhodobryum ontariense*	ZL^a^	1456/39°41’N114°37’E	110156X
4	Mniaceae	*Plagiomnium*	*Plagiomnium actum*	ZL^a^	981/39°49’N114°39’E	120043X
5			*Plagiomnium maximoviczii*	ZL^a^	988/ 39°49’N114°39’E	110136X
6		*Mnium*	*Mnium laevinarve*	ZL^a^	1267/39°43’N115°40’E	110052X

^a^ZL: Zhuolu county, Hebei province.

### Sample preparation

To avoid problems caused by the effect of water on FTIR spectra, all samples were dried at 45°C for 72 h, ground into fine powder in an agate mortar, and sieved through 200 meshes (75 μm).

### Spectral measurements

The FTIR spectra (4000–600 cm^−1^, 4 cm^−1^ resolution, 32 scans) were obtained with a ZnSe-attenuated total reflectance (ATR) accessory (Pike Technologies, Madison, WI, USA), which was combined with an FTIR spectrometer Bruker Optics Vertex70 (Ettlingen Germany) and a DigiTect^TM^ detector, which can prevent external signal disturbance and guarantee the highest signal-to-noise ratio. After grinding, 6 mg of powdered samples was directly placed at approximately 2.54 mm^2^ on the center of the ZnSe crystal plate. All samples were pressed using an identical mechanical pressure, and the FTIR spectra were obtained. The FTIR spectra of the six samples of mosses were automatically baseline-corrected. The automatic baseline-corrected data of each sample was used for further analysis. Each sample was measured five times, and the averaged spectrum was obtained for the ATR-FTIR spectra analysis.

### Precision, repeatability, and stability test

Sample 1 (*Rhdobryum roseum*1) was used to validate the method. The precision test was conducted using replicate records (5×) of the same sample. The repeatability test was analyzed by gathering the data of five independently prepared samples of the same sample. The stability test was determined by five records of one sample solution in 24 h. The mean value relative standard deviation (RSD) of the absorbance and wave number represented by the common peaks of each test was calculated.

### Second-derivative transformation test

Second-derivative spectroscopy has been a common spectral analysis technique for decades [28–33]. Second-derivative spectroscopy enables more specific identification of small and nearby absorption peaks that are not resolved in the original spectrum, which offers a way to increase the specificity of absorption peaks for certain components of the species. Another obvious advantage of using the second derivatives is that the constant and linear components of baseline errors are removed in the differentiation [30], which increases the feasibility of the second-derivative spectroscopy for quantitative work. A second-derivative spectrum was calculated for each measured pixel using Savizkye-Golay algorithm (seven smoothing points). Further, representative chemical maps were calculated based on the derivative peaks.

### Data analysis

All replicate spectra from the 6 species were recorded and found to be in the range of 4000–600 cm^−1^. The spectral data were calculated using the OMINIC version 8 software (Thermo Fisher, Waltham, USA).

To further classify the spectra, different multivariate methods such as cluster analysis and PCA were applied after the second-derivative transformation test. Cluster analysis and PCA were performed using the IBM SPSS 19.0 software. The cluster analysis was used to sort the FTIR spectra into similar sets or groups. Satisfactory results on the FTIR spectra of complex biological molecules were obtained using Ward’s algorithm, Euclidean distances, or the correlation coefficient calculation as distance metrics [14,34]. In the present study, 6 samples (each had 5 replicates) were selected for clustering. To obtain more comprehensive and accurate data, we selected the absorption values in the range of 4000–600 cm^–1^ for the cluster analysis. The nearest-neighbor and Pearson correlations were used to construct the dendrogram.

PCA also provides information on the major spectral components, where dominant factors determine the differences among the samples [35,36]. PCA is used to extract the important features of a correlation matrix in terms of PCs. Only a few PCs are typically required to explain the majority of the observed variance. PCA can be used as a chemometric method for FTIR analysis. The analysis can be presented as two-dimensional (2D, two PCs) or three-dimensional (3D, three PCs) scatter plots [37]. The absorption values in the range of 4000–600 cm^−1^ were used for the PCA, and the factor loading was plotted.

FSD was performed using the OMINIC 8 software. DWT was performed using the MATLAB 7.1 software. Daubechies wavelet, which served as the analysis wavelet, can better explore the signal singularity [38]. FSD and one-dimensional stationary DWT were performed on different samples.

## Results and discussion

### FTIR analysis

**Fig 1:**
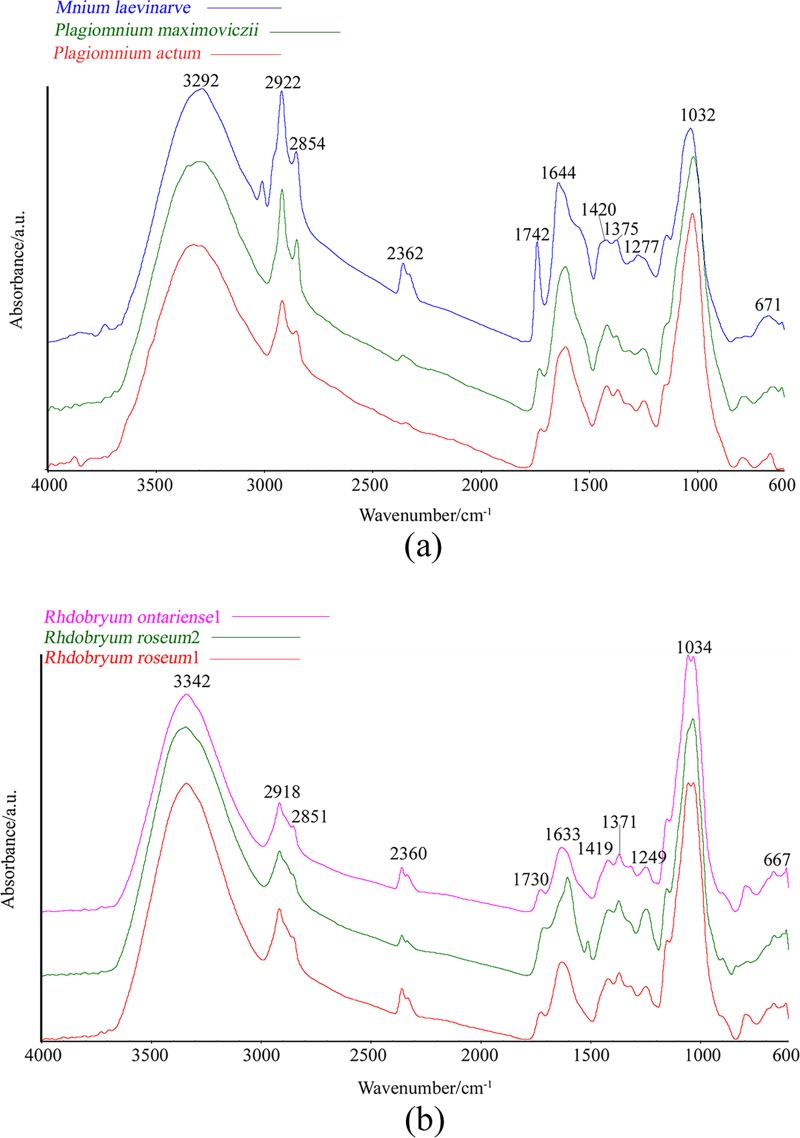
FTIR–ATR Spectra Obtained at 4000–600 cm^−1^ for Six Samples. The Main Peaks are Labeled at the Top of the Spectra.

The mean FTIR spectra of six moss samples were recorded and divided into two sets: three Mniaceae samples and three *Rhdobryum* samples (Fig 1). The characteristic peaks in the FTIR spectra from the six samples are shown in Fig 1 Most peaks represent major functional groups and show comprehensive information on the protein, carbohydrate, fibrin, and lipid components of the samples [12]. The FTIR spectra comparison can provide information about different samples. Hence, the region of 3500–3000 cm^−1^ presents a broad band centered at approximately 3300 cm^−1^, which corresponds to the absorption because of the stretching of the O–H and N–H bands (Fig 1). A sharp peak at 2920 cm^−1^ and 2850 cm^−1^ was attributed to the presence of polysaccharides, lipids, and carbohydrates (C–H stretch). The peak at 1640 cm^−1^ was attributed to the absorbance of amide (C = O bend). A second amide vibration appeared at 1420 cm^−1^ (C–H stretch), and an amide peak appeared at 1370 cm^−1^ (C–H stretch). The peak at 1030 cm^−1^ can be attributed to oligosaccharides, glycoprotein, and cellulose (C–O stretch) stretch. The peaks at 1300–600 cm^−1^ can be attributed to the absorbance of low-molecular-weight carbohydrates, polyols, and monosaccharides. This region is characterized as the fingerprint region [39].

### Second-derivative transformation

**Fig 2:**
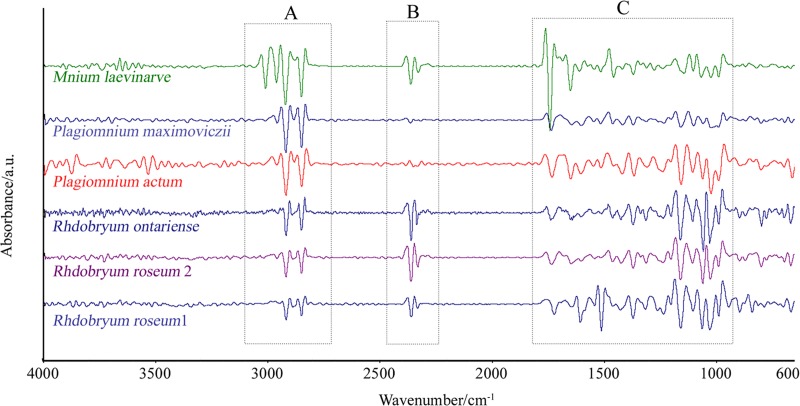
Mean Second-Derivative Spectra of Six Samples.

The mean second-derivative spectra of the 6 samples are compared in Fig 2. No significant difference in peak location was found in the investigated spectral region, except for the narrow peaks at approximately 2800 cm^−1^ (C-H stretch), 2400 cm^−1^(C = O stretch) and the region of 800–1700 cm^−1^ caused by Amide I II, C-N stretch, and N-H band. Although *Mnium laevinarve*, *Plagiomnium actum* and *P*. *maximoviczii* can be differentiated from the species of genus *Rhdobryum*, the differences between *Rhdobryum roseum* and *Rhdobryum ontariense* are not obvious after the second-derivative transformation (Fig 2).

### Cluster analysis

The FTIR spectra from different species of mosses exhibit similar absorbances. The second-derivative transformation test cannot provide obvious differences between *Rhodobryum roseum* and its adulterants. Specific differences are also difficult to distinguish by experience. Therefore, we used multivariate statistical methods to analyze the absorption bands. A cluster analysis was conducted to investigate the relationship among the species. We selected the absorption values in the range of 4000–600 cm^−1^. The nearest neighbor was used to construct the dendrogram according to the absorption values.

**Fig 3:**
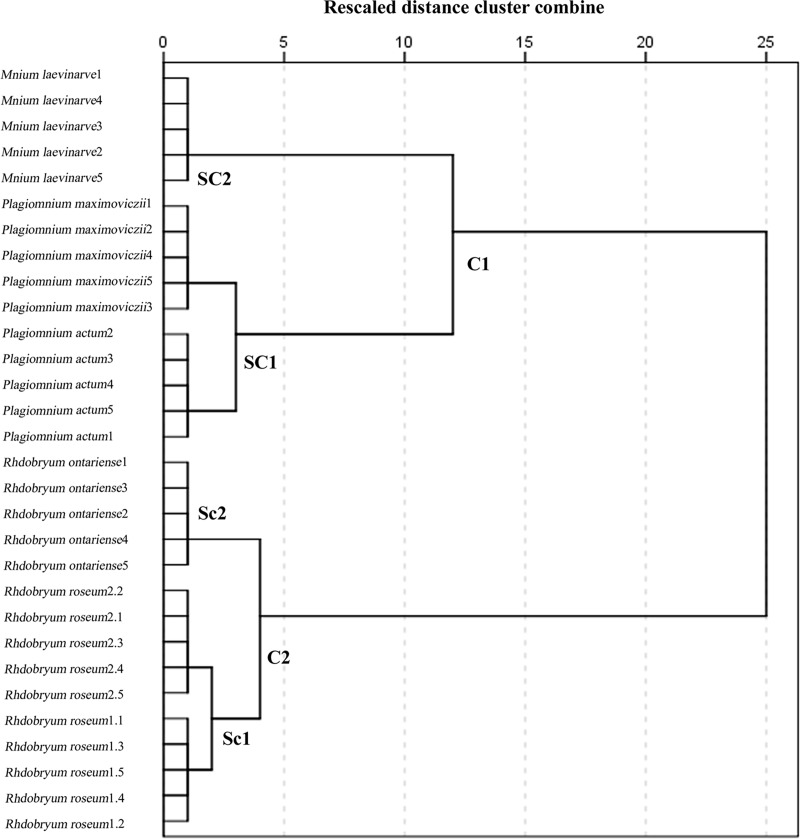
Dendrogram of the Relationship between 6 Moss Samples (5 Replicates for Each Sample). The dendrogram clustered by the hierarchical cluster analysis based on the FTIR data.

The dendrogram divides the six species (5 replicates for each sample) into two separated clusters (Fig 3): cluster 1 (C1) has three species from the family Mniaceae, and cluster 2 (C2) has three species from genera *Rhodobryum*. C1 is subdivided into two secondary sub-clusters. Sub-cluster 1 (SC1) is composed of 5 replicate samples of *Plagiomnium actum* and 5 replicate samples of *P*. *maximoviczii*. Sub-cluster 2 (SC2) has 5 replicate samples from the genera *Mnium* (Family Minaceae). The two sub-clusters were clustered together, which is consistent with the traditional morphologic classification. In C2, the three species of genus *Rhodobryum* were clustered together, and 5 replicate samples of *Rhodobryum roseum* 1 and 5 replicate samples of *Rhodobryum roseum* 2 were clustered together (Sc1). The replicate samples of *Rhodobryum ontariense* were clustered separately (Sc2). The replicate samples of each species were hardly discriminated.

The cluster analysis result indicates that *Rhodobryum roseum* is closely related to *Rhodobryum ontariense*. The phylogenetic relationship of the family Mniaceae is distant from genera *Rhodobryum*. As expected, the two main groups (genera *Rhodobryum* and family Mniaceae) are completely divided into two parts. However, the same species collected from different areas and the replicate samples of each species can hardly be distinguished by the cluster analysis.

The results can basically reflect the relative relationship among the 6 species. Further study is required to identify the internal relationships of the genera and the species collected from different areas. PCA, FSD, and one-dimensional DWT were used in our study.

### PCA analysis

**Fig 4:**
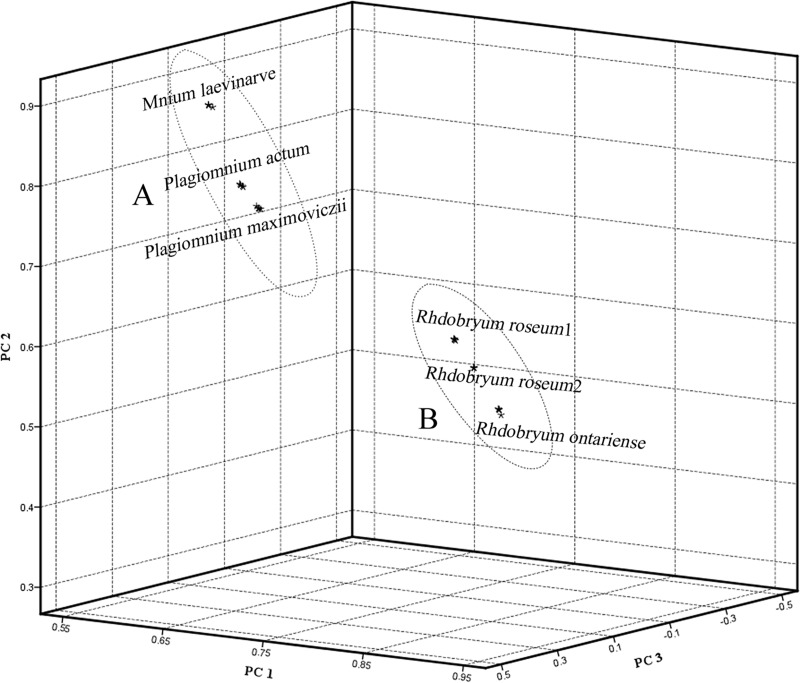
3D Plot of FTIR Spectra of the 6 Species of Mosses (5 Replicates for Each Sample) Based on PCA.

In the present study, we used PCA as the second multivariate analysis. The data from the absorption values in the range of 4000–600 cm^−1^ in the FTIR spectra were analyzed by PCA. Table 2 shows the variance that accounts for the first four PCs computed from the absorbance value of the characteristic peaks in the cluster analysis. The first three PCs summarized more variation in the data than in any other PC and accounted for more than 99.73% of the data variance. Fig 4 shows the score plot based on the first three PCs. The score plot indicates that the species of family Mniaceae and genera *Rhodobryum* can be grouped into two separate ellipses (A and B, Fig 4). The score plots of the replicate samples of each species are mostly overlapped. There is nearly no variation between the replicate samples of the same species. The three studied *Rhodobryum* species (*Rhdobryum ontariense*, *Rh*. *roseum* 1, *Rh*. *roseum* 2) formed one well supported group (Fig 4). The *Plagiomnium* species exhibits a short-distance relationship with *Mnium laevinerve*. The cluster analysis and PCA data suggest that the species of genus *Plagiomnium* (*Plagiomnium actum*, *P*. *maximoviczii*) is more closely related to *Mnium laevinerve* (Fig 4). In the traditional morphologic classification system, the two genera belong to the same family. The genus *Rhodobryum* belongs to the family Bryaceae. The PCA results are consistent with the cluster analysis results. The cluster analysis and PCA results can be used to simply, rapidly, and accurately identify different families and genera.

**Table 2:** Variance Distribution of the First Three PCs of the Six Moss Data Subsets.

PC	Variance %	Cumulative variance %
PC1	51.136	51.136
PC2	47.707	98.842
PC3	0.886	99.728

### FSD and wavelet analysis of the FTIR spectral data

The species of the same genus contain similar chemical components (e.g., protein, carbohydrate, and plant hormones) [36]. The FTIR spectra of the same genus exhibit close absorbance values and nearly identical wave numbers. In particular, the FTIR absorptions of the species from different places are difficult to distinguish. Although the cluster analysis and PCA can basically discriminate different families and genera, the differences between the same species and the replicate samples are barely visible. Therefore, in our study, FSD and DWT were used to extract the FTIR spectra features for further identification. The 1800–600 cm^−1^ region can provide higher characteristic molecular structural information on the spectra. The 1800–600 cm^−1^ fingerprint region contained greater molecular structural information and was used for the FSD and DWT analysis.

### FSD analysis

According to the classical taxonomy of bryophytes, genus *Rhodobryum* belongs to the family Bryaceae, whereas genera *Plagiomnium* and *Mnium* belong to the family Mniaceae. The common spectral peaks and absorbance values of the three species are not easy to classify by experience. FSD can be used to distinguish the small differences of different genera. Although it does make the signal peaks narrower, the FSD does not change the position or area of the peaks. Therefore, we applied the FSD method on 6 species of *Rhodobryum ontariense*, *Rh*. *roseum* 1, *Rh*. *roseum* 2, *Plagiomnium actum*, *P*. *maximoviczii* and *Mnium laevinarve*. The 1800–600 cm^−1^ range includes the fingerprint region, which contains more molecule structure information. There is more information for the range 1800 to 600 cm^−1^. Therefore, we use this region to extract spectral features.

**Fig 5:**
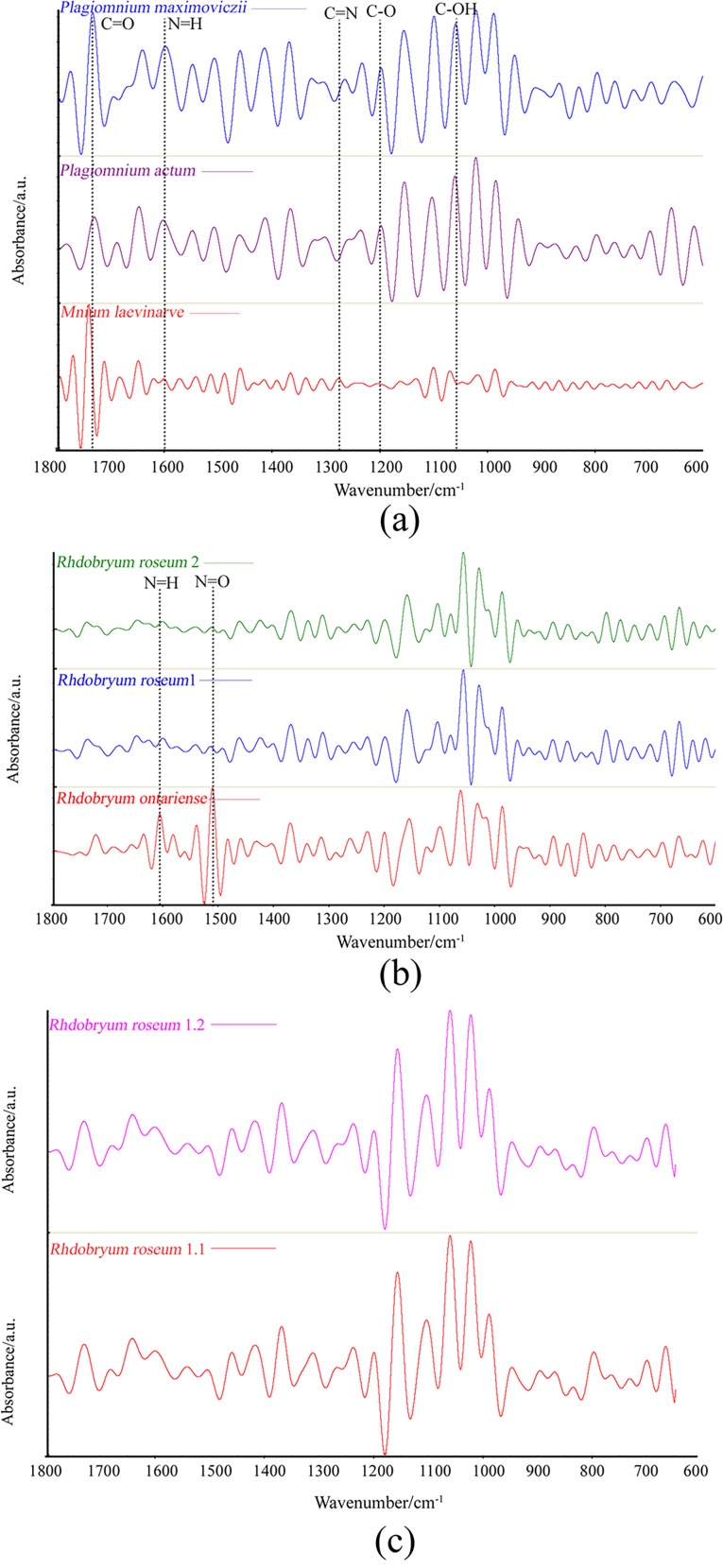
FSD FT-IR Spectra in the 1 800–600 cm^−1^ Region in the Whole Plants of the 6 Samples. The characteristic absorption bands are indicated at the top.

The FTIR–FSD spectral results in the region of 1800–600 cm^−1^ are displayed in Fig 5, which shows that the output waveforms of all 6 species have a marked variation. Certain differences are observed among *Plagiomnium actum*, *P*. *maximoviczii* and *Mnium laevinarve* at the identical resolution degree. According to Fig 5A, the C-O bend at 1205 cm^–1^ was found in *Plagiomnium actum* and *P*. *maximoviczii* but not *Mnium laevinarve*. The absorption bands at 1715 cm^–1^ (C = O), 1600 cm^–1^ (N = H), 1300 cm^–1^ (C = N), 1210 cm^-1^ (C-O), and approximately 1060 cm^–1^ (C-OH) have different shapes. Thus, FSD can be used to classify different species of indifferent genera of the same family and different species of the same genus. Then, we used FSD to identify *Rhodobryum roseum* (*Rhodobryum roseum*1, *Rhodobryum roseum*2) and *Rh*. *ontariense*, both of which belong to the genus *Rhodobryum*. The three species contain similar chemical components (e.g., amino acids, proteins, stigmasterol, and friedelin [40]). The FTIR–FSD spectra of the three species of genus *Rhodobryum* are shown in Fig 5B. However, the outcome is unsatisfactory. The absorption bands at 1600 cm^-1^ (N = H) and 1500 cm^-1^ (N = O) in *Rhodobryum roseum* and *Rh*. *ontariense*, have obviously different shapes, but the bands at 1500–600 cm^-1^ are similar. In particular, the two species of *Rhodobryum roseum* collected from different areas (*Rhodobryum roseum*1 and *Rhodobryum roseum*2) are almost identical (Fig 5C). The FSD spectra of the replicate samples of *Rhodobryum roseum* are nearly identical.

### DWT analysis

**Fig 6:**
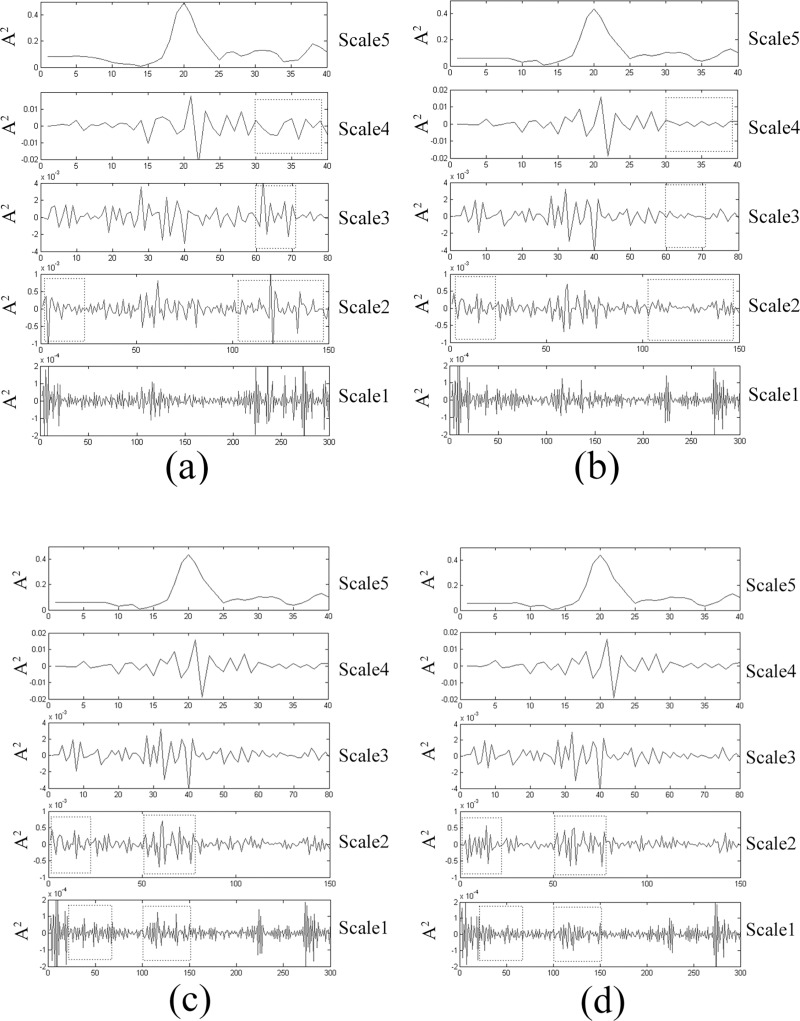
Results of the Multi-Resolution Decomposition for the FTIR Spectra with DWT. *Rhodobryum roseum* collected from different areas (a): *Rhodobryum roseum* 1; (b): *Rhodobryum roseum* 2; Two Replicates of *Rhodobryum roseum*; (c): *Rhodobryum roseum* 2.1; (d): *Rhodobryum roseum* 2.2.

Two samples (*Rhodobryum roseum*1 and *Rh*. *roseum*2) were collected from different areas. The chemical components of the two samples of *Rhodobryum roseum* are not significantly different, and the FTIR spectra of the two samples were difficult to differentiate. The FSD spectra of three samples of genus *Rhodobryum* are considerably similar to one another (Fig 5B). To further identify the two *Rhodobryum roseum* samples of the same species and the replicate samples, DWT was used to clarify the FTIR spectra. Scales 1–5 present the detailed information after decomposition (Fig 6). Five scales were compared. One-dimensional DWT was applied to decompose the FTIR spectral data of the two samples into different frequency bands. The vibration signals were decomposed up to five levels by using the Daubechies 4 mother wavelet. The DWT coefficients effectively reflected the features of the spectra (Fig 6). Scale 1 contains substantial noise, which is unsuitable in analyzing the differences among the same species. Determining the differences in Scale 5 is also difficult. Scales 2–4 present the differences between *Rhodobryum roseum*1 and *Rh*. *roseum*2 (Fig 6). Therefore, decomposition levels 2–4 in the DWT domain were selected as a variable characteristic extraction region to show the intraspecific variation of *Rhodobryum roseum*1 and *Rh*. *roseum*2. The results prove that DWT can also be used to extract the features of the FTIR absorptions of different samples of the same species. The replicate samples are quite difficult to distinguish by using all methods, which include the second-derivative transformation, cluster analysis, PCA and FSD. The DWT coefficients of the replicate samples (*Rhodobryum Roseum*2.1 and *Rhodobryum Roseum* 2.2) were found to have some differences in scales 1 and 2 (Fig 6). DWT can be used to identify the replicate samples of *Rhodobryum Roseum* cl. The result further proves that different closely related species can be easily distinguished by DWT.

### Validation of the method

#### Precision test

The precision test was conducted by obtaining replicate measurements of the same sample (*Rhodobryum roseum*) five times in an hour. The RSD of the FTIR absorbance value of the common peaks was ≤ 0. 92% (S1 Table).

#### Repeatability test

The repeatability of the method was assessed by analyzing five independently prepared samples of the same species (*Rhodobryum roseum*) using the identical method. The RSD of the FTIR absorbance value of the common peaks was ≤ 3.76% (S1 Table).

#### Sample stability test

The sample stability was determined using *Rhodobryum roseum* as an example. The same sample solution was analyzed at 0, 4, 8, 16, and 24 h after preparation. The RSD of the FTIR absorbance value of the common peaks were ≤ 3.47% (S1 Table). The similarity of the results indicates that this sample remained stable for 24 h. The running results show that the FTIR absorbance value of the characteristic common peaks of the same species is stable. Therefore, the method is reliable, exhibits good repeatability, and can be applied in the analysis of other moss samples.

## Conclusion

In recent years, *Rhodobryum roseum* has received increasing interest among researchers [41]. Discriminating *Rhodobryum roseum* from its adulterants remains difficult. The traditional circumscriptions are mainly based on the characteristics of the leaf cells, which makes the classification of the taxa difficult. The results of the present study show that FTIR spectroscopy with PCA and cluster analyses can be used to discriminate *Rhodobryum roseum* from other adulterants. In our study, we find that the cluster analysis and PCA can basically classify the species into groups. FSD is applied to extract the features and enhance the differences among the species from different genera with similar FTIR spectra. FSD with DWT can successfully identify *Rhodobryum roseum* species from different areas and replicate samples of the same species. Thus, the FTIR spectroscopy method with DWT is suitable for discriminating different species of mosses from controversial groups. The results show the possibility of using optical methods such as the FTIR method to differentiate the genera and species of mosses. The use of FTIR with chemometric methods to identify mosses is a rapid and efficient technique that can enable routine laboratories to facilitate the identification of mosses.

## Supporting information

S1 Table The automatic baseline-corrected data of 6 samples and the Precision test, Repeatability test, Sample stability test data. (XLSX)

## References

[1] Crosby MR, Magill RE, Allen B, He S. A checklist of the mosses [Internet]. St Louis: Missouri Botanical Garden; 1999 [cited 2013 May15]. Available from: http://wwwmobotorg/MOBOT/tropicos/most/checklistshtml.

[2] Foan L, Leblond S, Thöni L, Raynaud C, Santamaría JM, Sebilo M, et al Spatial distribution of PAH concentrations and stable isotope signatures (δ13C, δ15N) in mosses from three European areas–Characterization by multivariate analysis. Environ Pollut. 2014;184: 113–122. 10.1016/j.envpol.2013.08.006 24047547

[3] Crandall-Stotler B, Bartholomew-Began S. Morphology of mosses (Phylum Bryophyta) In: Flora of North America Editorial Committee, editors. Flora of North America North of Mexico. New York & Oxford: Flora of North America North of Mexico; 2007 pp. 3–13.

[4] Cox CJ, Hedderson TAJ. Phylogenetic relationships among the ciliate arthrodontous mosses: evidence from chloroplast and nuclear DNA sequences. Plant Syst Evol. 1999;215: 119–139.

[5] Cox CJ, Hedderson TAJ. Phylogenetic relationships within the moss family Bryaceae based on chloroplast DNA evidence. J Bryol. 2003;25: 31–40.

[6] Cox CJ, Goffinet B, Shaw AJ, Boles SB. Phylogenetic relationships among the mosses based on heterogeneous bayesian analysis of multiple genes from multiple genomic compartments. Syst Bot. 2004;29: 234–250.

[7] Montes J, Jimenez J, Cano M, Jiménez-Martínez J. A contribution to the phylogenetic study of Mielichhoferiaceae-Mniaceae (Bryophyta) based on molecular sequence data. Nova Hedwigia. 2011;93: 47–56.

[8] Cheng C, Liu J, Cao W, Zheng R, Wang H, Zhang C. Classification of two species of Bidens based on discrete stationary wavelet transform extraction of FTIR spectra combined with probability neural network. Vib Spectrosc. 2010;54: 50–55.

[9] Cheng C-G, Tian Y-M, Zhang C-J. Research of recognition method between Semen cuscutae and its sibling plant Japanese dodder seed based on FTIR-CWT and ANN classification method. Acta Chim Sin. 2008;66: 793–798.

[10] Zhang C-J, Cheng C-G. Identification between Stephania tetrandra S. Moore and Stephania cepharantha Hayata by CWT–FTIR–RBFNN. Spectroscopy. 2008;22: 371–386.

[11] Lu H, Cheng C-G, Tang X, Hu Z-H. FTIR spectrum of Hypericum and Triadenum with reference to their identification. Acta Bot Sin. 2004;46: 401–406.

[12] Hu T, Jin W-Y, Cheng C-G. Classification of five kinds of moss plants with the use of fourier transform infrared spectroscopy and chemometrics. Spectroscopy. 2011;25: 271–285.

[13] Naumann A. A novel procedure for strain classification of fungal mycelium by cluster and artificial neural network analysis of fourier transform infrared (FTIR) spectra. Analyst. 2009;134: 1215 10.1039/b821286d 19475151

[14] Naumann D, Helm D, Labischinski H, Schallehn G. Classification and identification of bacteria by fourier-transform infrared spectroscopy. J Gen Microbiol. 1991;137: 69–79. 10.1099/00221287-137-1-69 1710644

[15] Wenning M, Scherer S. Identification of microorganisms by FTIR spectroscopy: perspectives and limitations of the method. Appl Microbiol Biotechnol. 2013;97: 7111–7120. 10.1007/s00253-013-5087-3 23860713

[16] Wang X, Chen X, Qi Z, Liu X, Li W, Wang S. A study of Ganoderma lucidum spores by FTIR microspectroscopy. Spectrochim Acta A Mol Biomol Spectrosc. 2010;91: 165–178. 10.1016/j.saa.2012.02.004 22381804

[17] Movasaghi Z, Rehman S, Rehman DI. Fourier transform infrared (FTIR) spectroscopy of biological tissues. Appl Spectrosc Rev. 2008;43: 134–179.

[18] Chan KLA, Kazarian SG. Attenuated total reflection–fourier transform infrared imaging of large areas using inverted prism crystals and combining imaging and mapping. Appl Spectrosc. 2008;62: 1095–1101. 10.1366/000370208786049042 18926018

[19] Poulli KI, Mousdis GA, Georgiou CA. Classification of edible and lampante virgin olive oil based on synchronous fluorescence and total luminescence spectroscopy. Anal Chim Acta. 2005;542: 151–156.

[20] de Luca M, Terouzi W, Ioele G, Kzaiber F, Oussama A, Oliverio F, et al Derivative FTIR spectroscopy for cluster analysis and classification of morocco olive oils. Food Chem. 2011;124: 1113–1118.

[21] Naumann A, Heine G, Rauber R. Efficient discrimination of oat and pea roots by cluster analysis of Fourier transform infrared (FTIR) spectra. Field Crops Res. 2010;119: 78–84.

[22] Forina M, Oliveri P, Lanteri S, Casale M. Class-modeling techniques, classic and new, for old and new problems. Chemometr Intell Lab Syst. 2008;93: 132–148.

[23] Salzer R, Mantsch HH, Mansfield J, Lewis EN, Steiner G. Infrared and raman imaging of biological and biomimetic samples. Fresenius J Anal Chem. 2000;366: 712–726. 11225782 10.1007/s002160051565

[24] Daubechies I. The wavelet transform, time-frequency localization and signal analysis. IEEE Trans Inf Theory. 1990;36: 961–1005.

[25] Marchant BP. Time–frequency analysis for biosystems engineering. Biosystems Eng. 2003;85: 261–281.

[26] Subasi A. Medical decision support system for diagnosis of neuromuscular disorders using DWT and fuzzy support vector machines. Comput Biol Med. 2012;42: 806–815. 10.1016/j.compbiomed.2012.06.004 22763356

[27] Addison P, Sibbald A, Watson J. Wavelet analysis: a mathematical microscope with civil engineering applications. Insight. 1997;39: 493–497.

[28] Dong A, Huang P, Caughey WS. Protein secondary structures in water from second-derivative amide I infrared spectra. Biochemistry. 1990;29: 3303–3308. 2159334 10.1021/bi00465a022

[29] Giese AT, French CS. The analysis of overlapping spectral absorption bands by derivative spectrophotometry. Appl Spectrosc. 1955;9: 78–96.

[30] Kohler A, Bertrand D, Martens H, Hannesson K, Kirschner C, Ofstad R. Multivariate image analysis of a set of FTIR microspectroscopy images of aged bovine muscle tissue combining image and design information. Anal Bioanal Chem. 2007;389: 1143–1153. 10.1007/s00216-007-1414-9 17639358

[31] Mark H, Workman J. Derivatives in spectroscopy: part 2 –The “true” derivative. Spectroscopy. 2003;18: 25–28.

[32] Susi H, Byler D. Protein structure by fourier transform infrared spectroscopy: second derivative spectra. Biochem Biophys Res Commun. 1983;115: 391–397. 6615537 10.1016/0006-291x(83)91016-1

[33] Whitbeck MR. Second derivative infrared spectroscopy. Appl Spectrosc. 1981;35: 93–95.

[34] Naumann D, Fijala V, Labischinski H, Giesbrecht P. The rapid differentiation and identification of pathogenic bacteria using fourier transform infrared spectroscopic and multivariate statistical analysis. J Mol Struct. 1988;174: 165–170.

[35] Defernez M, Kemsley EK, Wilson RH. The use of FTIR and chemometrics for the authentication of fruit purees. J Agric Food Chem. 1995;43: 109–113. 10.1021/jf981196d 10552633

[36] Kemsley EK, Belton PS, McCann MC, Ttofis S, Wilson RH, Delgadillo I. A rapid method for the authentication of vegetable matter using fourier transform infrared spectroscopy. Food Control. 1994;5: 241–243.

[37] Sockalingum GD, Bouhedja E-W, Allouch P, Bloy C, Manfait M. FT-IR spectroscopy as an emerging method for rapid characterization of microorganisms. Cell Mol Biol. 1998;44: 261–269. 9551657

[38] Yang J, You X, Tang YY, Fang B. A water marking scheme based on discrete non-separable wavelet transform. Image Anal. 2005;3522: 427–434.

[39] Asakawa Y, Ludwiczuk A, Nagashima F. Phytochemical and biological studies of bryophytes. Phytochemistry. 2013;91: 52–80. 10.1016/j.phytochem.2012.04.012 22652242

[40] Xu H-C, Zhou Y-B. Studies on chemical components and their pharmacological effects of Rhodobryum roseum Limpr. J Shaanxi Normal Univ. 2008;36: 88–92.

[41] Qiao F, Ma S, Lin R. Summarization of phytochemical and pharmacological research of grandifoliate moss. Drug Stanoaros of China. 2003;6: 3–5.

